# Characterization of *Xanthomonas arboricola* pv. *juglandis* Bacteriophages against Bacterial Walnut Blight and Field Evaluation

**DOI:** 10.3390/v14071380

**Published:** 2022-06-24

**Authors:** Julio Retamales, Pablo Núñez, Romina Alvarado, Erick D. M. Campan, Thierry Otto, Cristopher Segovia, Ignacio Vasquez, Javier Santander

**Affiliations:** 1 Instituto de Ciencias Naturales, Facultad de Medicina Veterinaria y Agronomía, Universidad de las Américas, Viña del Mar 2520000, Chile; 2 Agroadvance SpA, Peñaflor 9750000, Chile; pnunez@agroadvance.cl (P.N.); ralvarado@agroadvance.cl (R.A.); 3 Laboratoire Écologie Fonctionnelle et Environnement, Université de Toulouse, CNRS, 31062 Toulouse, France; erick.campan@univ-tlse3.fr (E.D.M.C.); thierry.otto@univ-tlse3.fr (T.O.); 4 Marine Microbial Pathogenesis and Vaccinology Laboratory, Department of Ocean Sciences, Memorial University of Newfoundland, St. John’s, NL A1C 5S7, Canada; cwsegovia@mun.ca (C.S.); ivasquezsoli@mun.ca (I.V.); jsantander@mun.ca (J.S.)

**Keywords:** *Xanthomonas arboricola* pv. *juglandis*, phage therapy, walnut blight

## Abstract

*Xanthomonas arboricola* pv. *juglandis* (hereafter *X. juglandis*) is the etiological agent of walnut blight, the most important bacterial disease affecting walnut production worldwide. Currently, the disease is treated mainly with copper-derived compounds (e.g., CuSO_4_) despite the evidence of genetic resistance in these strains. Regarding the effectiveness and sustainability, the use of a bacteriophage appears to be a biocontrol alternative to reduce *X. juglandis* load and symptomatology of walnut blight. Here, the phages *f*20-Xaj, *f*29-Xaj, and *f*30-Xaj were characterized, and their effectiveness in walnut orchards against walnut blight was determined. These bacteriophages showed a specific lytic infection in *X. juglandis* strains isolated from Chile and France. Phylogenetic analysis of the complete genome of *f*20-Xaj and *f*30-Xaj indicates that these phages belong to the *Pradovirus* genus. In the field, the cocktail of these bacteriophages showed similar effectivity to CuSO_4_ in the reduction of incidence and severity in walnut tissue. Moreover, the bacterial load of *X. juglandis* was significantly reduced in the presence of bacteriophages in contrast to a CuSO_4_ treatment. These results show that the use of bacteriophages can be an alternative to combat the symptoms of walnut blight caused by *X. juglandis*.

## 1. Introduction

Walnut blight is the main disease affecting walnut production globally [1]. Walnut blight is caused by the Gram-negative bacteria *Xanthomonas arboricola* pv. *juglandis* (hereafter *X. juglandis*) [2]. *X. juglandis* can infect flowers, shoots, leaves, and buds, where major economic losses are associated with necrotic damage to immature fruits [3]. This pathogen accesses walnut tissue by natural openings such as stomata and damaged tissues, preferably in wet springs, where up to 80% of the production can be lost [4].

The current control of *X. juglandis* is through the utilization of copper-based agrochemicals [5]. The efficiency of copper-base agrochemicals has decreased, due to the emergence of *X. juglandis-*resistant strains [6,7,8]. Recently, genomic studies showed that the *X. juglandis* genome had several genes related to copper resistance mechanisms [9,10,11,12]. Additionally, the utilization of heavy metals in a high dose, such as copper, during the production process has serious agronomic and sanitary implications, which results in rejection by consumers [13]. The utilization of bacteriophages (or phages) is an accessible and eco-friendly alternative to biocontrol phytopathogenic bacteria [14]. Recently, several studies have summarized the use of bacteriophages as a green solution for the control of plant diseases caused by *Xanthomonas* species [15]. This review describes the effectiveness of some bacteriophages in the control of diseases caused by *Xanthomonas* species. These evaluations carried out under greenhouse and/or field conditions show the effectiveness of the use of bacteriophages compared to conventional chemical treatments in minor plants (pepper, tomato, onion, and rice) and some fruit trees (peach seedlings and citrus). However, information about the bacteriophages’ performance to control *X. juglandis* in the field is needed.

The first isolated bacteriophages against different *X. juglandis* strains were obtained from soil samples of walnut trees in New Zealand [16]. They reported that bacteriophages tolerated different storage conditions, but their performance as a biocontrol for *X. juglandis* in a greenhouse environment or field trial was not successful. Subsequently, these researchers isolated twenty-six bacteriophages against *X. juglandis* from the phyllosphere and rhizosphere of walnut plant tissues obtained from different locations in New Zealand [17]. These authors provided a deeper description of bacteriophages, including sequencing fragments of the genetic material of these bacterial viruses. Although these authors showed that these bacteriophages have high host-specificity and considerable stability under specific storage conditions, the authors did not describe their biocontrolling capabilities against walnut blight in detail. Years later, two polyvalent phages against *X. juglandis* were isolated from infected walnut fields in Hungary [18]. These phages belong to the *Siphoviridae* and *Podoviridae* families, respectively, and were characterized through morphological, physiological, and genomic analyses. Both phages showed a significant lytic effect in vitro bacterial challenge assays. These authors deepened their studies on the genetic analysis of these two bacteriophages as they indicated that a detailed characterization of phage features and phage–host interactions is essential to evaluate the potential usage of phages as biocontrol agents.

In a previous study, we sequenced and annotated the genome of three lytic *X. juglandis* bacteriophages [19]. We found that these bacteriophage genomes were coding sequences for replication, virion structure, and cell endolysis. Furthermore, *f*20-Xaj and *f*30-Xaj possess RNA polymerase, suggesting that they are related to T7 phages [20]. The aim of this study was to provide a biological and genomic characterization of these *X. juglandis* bacteriophages isolated from infected walnut trees in Chile. In addition, we reported the application of these bacteriophages in field trials, comparing them to conventional agrochemical treatments. Our results suggest that the use of *f*20-Xaj, *f*29-Xaj, and *f*30-Xaj bacteriophage cocktails is a promising strategy to combat walnut blight.

## 2. Materials and Methods

### 2.1. Isolation of X. juglandis

A total of 115 colonies of yellow pigmentation were obtained from symptomatic samples of walnut leaves and immature fruits, all collected between 2014 and 2018 from different locations of walnut orchards in Chile and France. These isolates were routinely cultivated in YPG broth (10 g peptone; 5 g yeast extract; 5 g glucose per liter) or YPG supplemented with 1.5% agar for 48 h at 28 °C ± 0.5. The identification of each isolate as *X. juglandis* was initially performed by standard biochemical and physiological tests [1]. The pathogenicity of these bacterial isolates was confirmed in immature walnut fruits [21]. For comparative purposes, the isolate *X. juglandis* J303 was included in all assays [11]. Purified colonies identified as *X. juglandis* were kept at −80 °C in a mixture of glycerol (20% *v*/*v*) and YPG broth.

### 2.2. Identification of X. juglandis

DNA extraction from selected isolates was performed with the Wizard^®^ Genomic DNA Purification Kit (Promega, Madison, WI, USA), and used for 16S amplification using the universal primers EUBA (5′-AGAGTTTGATCMTGGCTCAG-3′) and EUBB (5′-AAGGAGGTGATCCANCCRCA-3′) [22], *Xanthomonas* genus-specific primers X1 (5′-AAGGATCGGGTATTAAC-3′) and X2 (5′-AGAGTTTGATCTTGGCTCAG-3′) [23], and *X. arboricola* species-specific primers XarbQ-F *qumA* (5′-GCGCGAGATCAATGCGACCTCGTC-3′) and XarbQ-R *qumA* (5′-GGTGACCACATCGAACCGCGCA-3′) [24], and were sequenced at Macrogen Inc. (Seoul, Korea).

### 2.3. Bacteriophages Isolation

Bacteriophages with lytic activity on *X. juglandis* were isolated from wet soil around walnut tree samples, including leaves, twigs, buds, staminate flowers, and immature fruit. These samples were homogenized or diluted in sterile PBS 1X and processed in enriched phages using a standard method [25]. Briefly, flasks containing 90 mL of YPG broth were inoculated with 0.5 mL of *X. juglandis* J303 in the exponential phase and 10 mL of the homogenized sample. The flask was placed on an orbital shaker at 150 rpm, and it incubated for 24 h at 28 ± 0.5 °C. The culture was pelleted by centrifugation at 6000× *g* for 10 min at 4 °C and the remaining bacteria in the supernatant was removed by filtering (0.22 µm) and combined with chloroform (1:20). Phages were detected in macroplaques using the double-agar-layer technique [26] and *X. juglandis* J303 as the host. The plaque was picked from the positive sample and re-plated three times to ensure clonal phage stocks.

### 2.4. In Vitro Propagation and Lytic Activity of Bacteriophage

Bacteriophages obtained were routinely propagated in YPG broth inoculated with fresh exponentially growing cultures of *X. juglandis* J303 (1:100). Cultures were infected at a multiplicity of infection (MOI) of 1 at 150 rpm and incubated at 28 °C until a minimum value of O.D._600nm_ was reached (~0.1). From these lysates, the bacteriophage was precipitated with the polyethylene glycol (PEG-8000) method [27]. For in vitro bacterial reduction experiments, 0.1 mL of an exponential culture of *X. juglandis* J303 was added to 100 mL of YPG broth. When the bacterial culture reached an O.D._600nm_ ≈ 0.1 (5 × 10^5^ CFU/mL) maintained at 28 °C with agitation (150 rpm), phages *f*20-Xaj, *f*29-Xaj, and *f*30-Xaj were added at 0.1 of MOI, and bacterial reduction was observed every 60 min in a spectrophotometer (Metertech SP-830, Taipei, Taiwan) by measurement of the optical density at 600 nm (O.D._600nm_). Each bacteriophage was tested in triplicate, and the bacterial culture without a bacteriophage added served as a control group.

### 2.5. One-Step Phage Growth Curve

One-step growth curves were performed as described previously [28] with modifications. Briefly, 5 mL of the exponential culture of *X. juglandis* J303 (10^8^ CFU/mL) and individual phage suspensions were mixed at MOI of 0.1 in 50 mL of YPG broth. The samples were incubated at 28 °C for 10 min to allow for phage adsorption, and then cultures were diluted 10,000-fold in YPG broth. Infected cells were incubated at 28 °C with constant shaking (150 rpm), and samples of 1 mL were taken every 10 min. The latent period was defined as the time interval between the adsorption (not including 10 min pre-incubation) and the beginning of the first burst indicated by the initial rise in phage titer [29]. The burst size was calculated as the ratio between the final count of liberated phage particles and the initial count of infected bacterial cells during the latent period [30]. The experiment was performed three times.

### 2.6. Host Range Determination

The three bacteriophages selected were tested against *X. juglandis* isolates and several bacterial strain isolates from the walnut tree. Bacterial identification was conducted by 16S rRNA gene sequencing using a universal primer EUBA and EUBB [21]. Each polymerase chain reaction (PCR) was carried out in a total volume of 25 µL containing 1 µL of each primer (5 µM), 12.5 µL of the GoTaq^TM^ Green Master Mix (Promega, Madison, WI, USA), and 1 µL of template DNA (20 ng/µL) in sterile water. PCR amplification was performed on a Scientific Mastercycler Personal thermocycler (Eppendorf, Framingham, MA, USA) using the following conditions: 5 min for initial denaturation at 94 °C, followed by 30 cycles of 94 °C for 30 s, 57 °C for 30 s, and 72 °C for 1 min, with a final extension step of 72 °C for 5 min. PCR products were separated and visualized in 1% agarose gel with ethidium bromide (0.5 µg/mL), and purified using a genomic DNA kit by ATP^TM^ Biotech according to the manufacturer’s instructions. DNA sequencing of the 16S rRNA PCR products was performed by Macrogen, Inc. (Seoul, Korea). DNA sequences were analyzed using MEGA5 software [31], and a homology search was performed with the nonredundant (nr) database of the NCBI (National Centre for Biotechnology Information, Bethesda, MD, USA) through BLASTn [32]. The host range of bacteriophages was determined using a spot test procedure [33]. Briefly, exponential phase cultures of *X. juglandis* J303 were grown in YPG broth and then sub-cultured with the addition of 100 μL to 3 mL of YPG broth and grown at 28 °C for 6 h. Thus, 100 μL of the sub-culture was inoculated into 3 mL of YPG soft agar (0.75% agar) and overlaid onto YPG agar plates until allowed to solidify for 15 min. Aliquots of 5 μL of every phage lysate (with titers of approximately 10^7^ PFU/mL) were spotted onto the bacterial overlay, dried, then incubated at 28 °C overnight. As a control, each bacterial strain was also mock-infected with a sterile phage buffer. Specific bacteriophage-sensitive isolates showed clear areas where the bacteriophage suspensions had been spotted and recorded on a descriptor as follows: No plaques, turbid plaques, or clear plaque.

### 2.7. Test for Lysogeny

This test was performed as described previously [34], with some modifications. Initially, an exponential growth phase *X. juglandis* J303 culture was employed to obtain a lawn on YPG agar. Aliquots of 50 μL from individual and mixtures of bacteriophage stock (1 × 10^8^ PFU/mL) were deposited in the center of a petri dish for generating a lytic macroplate. These plates were incubated at 28 °C for 72 h until the development of colonies in the bacteriophage spot zone. Each colony (30 per phage) was reisolated and purified from the phage culture after three successive rounds of seeding on the YPG plate agar. Supernatants from overnight cultures of this phage-resistant bacteria on YPG broth were collected and tested for the spontaneous release of phage particles by spotting 5 μL drops onto soft agar containing *X. juglandis* J303 bacterial hosts, and plates were examined for bacterial cell lysis phage infection, identified by zones of clearance. The supernatants from *E. coli* OP50 y *S. enteritidis* ATCC 13076 were used as negative controls, and pure phage solutions (10^8^ PFU/mL) were used as positive controls in a double-agar-layer assay by lysis plaque formation.

### 2.8. Stored Stability of Bacteriophages

In order to test bacteriophage stability, individual phages (1 × 10^5^ PFU/mL) were incubated on YPG broth at −20, 4, 17, and 28 °C, and phage titers were determined after 28 days of incubation using the double-layer agar technique and *X. juglandis* J303 as the host strain. Stability also was determined in distilled water, tap water, and YPG broth supplemented with algae extract and commercial doses of copper derived as cuprous oxide (2 g/L), copper hydroxide (2.5 g/L), and copper sulfate (3 g/L).

### 2.9. Transmission Electron Microscopy

*X. juglandis* J303 and phage preparations (20 μL of 2.5 × 10^10^ PFU/mL) were added to 300-Mesh copper grids coated with Formvar (Sigma-Aldrich, St. Louis, MI, USA) and negatively stained for 30 s with a water dilution of 1% uranyl acetate [35]. Once air-dried, the samples were examined on a Philips TECNAI 12 transmission electron microscope (TEM) operating at 80 Kv.

### 2.10. Nucleic Acid Extraction from Phages

The nucleic acid of phages was isolated according to Kaiser and collaborators [36], with some modifications. Briefly, 10 mL of crude phage suspension (10^9^ PFU/mL) was purified by filtration employing a syringe filter with 0.22 µm pore diameter, and 1 µL of DNAse I (10 mg/mL) was added. To 2 mL of purified phage suspension, 1 µL of RNAse (10 mg/mL) was added and the mix was incubated for 30 min at room temperature. After the enzyme treatment, 250 µL of 0.1 M EDTA (pH 7.5) and 4 µL of Proteinase K (20 mg/mL) were added and incubated for 15 min at 45 °C. Later, 25 µL of 5% Hexadecyltrimethylammonium bromide (CTAB, Merck, Darmstadt, Germany) was incubated for 3 min at 60 °C and cooled in ice for 5 min. It was then centrifuged to 10,000× *g* for 10 min and the supernatant was discarded. The pellet with nucleic acid was resuspended with 200 µL of 1.2 M NaCl and 0.5 mL of absolute ethanol was carefully added and incubated for 10 min at room temperature. Nucleic acid was centrifuged for 10 min at 10,000× *g* and washed with 0.5 mL 70% ethanol, air dried, and centrifuged again. The pellet was resuspended in ultrapure water and stored at −20 °C until use.

### 2.11. Phylogenetics and Comparative Genomics Analysis of Bacteriophages

Two phages with differential bacterial host range profiles (Table 1) were subjected to bioinformatic analysis. For this, previously, *f*20-Xaj and *f*30-Xaj genomes were sequenced through Illumina MiSeq and assembled using CLC Genomics Workbench 8.5.1 (Qiagen, Hilden, Germany). Both assembled genome sequences were annotated by the National Center for Biotechnology Information (NCBI, Bethesda, MD, USA) Prokaryotic Genomes Annotation Pipeline (PGAP) and deposited with GenBank accession numbers of KU595432 and KU595433, respectively [11]. The Neighbor-Joining method was used to infer the evolutionary history [37]. Bootstrap analysis was performed with 1000 resamplings. The evolutionary distances were computed using the p-distance method [38] and are in the unit of the number of amino acid differences per site. This analysis involved 20 amino acid sequences. All ambiguous positions were removed for each sequence pair (pairwise deletion option). There was a total of 419 positions in the final dataset. Phylogenetic analysis was performed using MEGAX [39]. The phage genomes utilized in this analysis were included according to viral morphology classification in *Pradovirus*: Pagan MK903278.1 (*Xanthomonas* sp.), phi Xc10 NC_047840.1 (*Xanthomonas* sp.), Xaj24 NC_047762.1 (*Xanthomonas* sp.); Prado NC_022987.1 (*Xylella* sp.), Titan-X MN478375.1.); *Autographiviridae*: Paz NC_022982.1 (*Xylella* sp.), Arno160 NC_048111.1 (*Pectobacterium* sp.), PP81 NC_047797.2 (*Pectobacterium* sp.), RLP NC_048168.1 (*Pseudomonas* sp.), Lidtsur NC_048177.1 (*Escherichia* sp.), vB_VpaS OwB NC_048167.1 (*Vibrio* sp.), S-B28 NC_048171.1 (*Synechococcus* sp.), RPSC1 NC_047982.1 (*Ralstonia* sp.), Katbat NC_048057.1 (*Dickeya* sp.), Mysterion NC_048060.1 (*Dickeya* sp.), GW1 NC_048161.1 (*Cronobacter* sp.), and VB_shipA7 NC_048180.1 (*Shigella* sp.). *Siphoviridae*: *f*18SE NC_028698.1 (*Salmonella* sp.) was included as an outgroup. A circular representation of the genome of both *f*20-Xaj and *f*30-Xaj phages was visualized using the CGView server database [40]. Comparative analysis of genome sequences and visualization between *f*20-Xaj and *f*30-Xaj genomes was performed using BLASTn and BLASTp databases and the EasyFig program (Version 2.2; Beatson Microbial Genomic Lab, Brisbane, Australia) [41]. Phage genomes were compared using the CLCBio whole-genome alignment tool, and alignment was computed using the following parameters: Min. seed length = 15 min. alignment block length = 100, allowed mismatches and rearrangement contigs. Average nucleotide identity (ANI) analysis was computed using a min. similarity fraction = 0.8, and a min. length fraction = 0.8. Dot plot analysis was performed using a min. seed length = 15 and allowed mismatches.

### 2.12. Field Trials against X. juglandis with Bacteriophages

With the aim of evaluating the effect of bacteriophage application on bacterial walnut blight appearance, an experimental field trial was conducted with the walnut Chandler variety in the Villa Alegre commune of the VII region of Chile between October 2015 and March 2016. This trial was carried out with permission given by Servicio Agricola y Ganadero (SAG-Chile) through the exempt resolution N°: 7898/2015 that considers the natural inoculum of *X. juglandis* employing fields with a historic presence of walnut blight. The treatments evaluated were arranged in a randomized complete block design with four replicates. Each experimental unit consisted of six trees, and two central trees were taken for evaluation. All treatments were applied six times during sunrise in approximately 7–8-day intervals. These walnut trees were maintained with the conventional approach of fertilization and irrigation. The phages *f*20-Xaj, *f*29-Xaj, and *f*30-Xaj were produced independently using 1 L of YPG broth as described above. After the incubation time, broths were filtered with 0.22 µm-pore-size membrane filters, and the phage concentration (PFU/mL) was determined as described above. Phage cocktails were applied to walnut trees using 2, 3, and 4 cc/L doses, employing a compression sprayer. The application of the different treatments was carried out six times. For comparative purposes, two control groups were included. One of these groups of walnut trees had water applied (UTC) and the second group was treated with commercial copper sulfate as a standard dose program (CuT) (six applications at doses of 3 g/L). The efficiency of phage applications was determined using an incidence and severity index as well as the production performance of walnut nuts (Kg) after trials finished. The incidence was determined by counting the occurrence of symptomatic walnut blight in leaves/fruits from the total number of samples collected in each group, according to the protocol established by the Commonwealth Mycological Institute (CAB). The severity index was determined using a scale of 1 to 4 based on the visual appearance of the area of damage in leaves and fruit, where 1 = healthy tissue, 2 =< 1–25% of leaves/fruit affected, 3 = 26–50% of leaves/fruits affected, and 4 > 50% of leaves/fruits affected. Production performance was determined after randomly weighing forty fruits per tree from each treatment. Additionally, the bacterial load (CFU/g) present on walnut fruits was subjected to determination. For this purpose, 10 immature walnut fruits were collected from different sectors of each tree of the different treatments. Each fruit was macerated and weighed individually and by serial dilutions to determine the load of *X. juglandis* in the YPG medium. In each instance of evaluation, visual aspects of phytotoxicity from walnut tree treatments with phages were determined.

## 3. Results

### 3.1. Isolation and Identification of X. juglandis

A total of fourteen bacterial isolates were obtained from symptomatic walnut fruit and identified as *X. juglandis* by sequencing 16S rRNA. In addition, all bacteria identified as *X. juglandis* were able to generate necrotic damage on immature walnut fruits and were used to host a range of assays (Table 1). For the bacteriophage isolation, the strain denominated *X. arboricola* pv. *juglandis* J303 was employed [11]. This isolate is rod-shaped, 0.5 × 2.5 µm in size, surrounded by a dense polymeric matrix, and able to develop robust damage in the in vitro walnut fruit pathogenicity assay (Figure S1). *X. juglandis* J303 possess homogeneous growth in broth media, facilitating bacteriophages assays. Furthermore, *X. juglandis* J303 also has a biochemical profile that is characterized by an oxidative enzymatic profile and low use of carbon sources established in an API test. *X. juglandis* J303 has a highly susceptible profile to antibiotics commonly used in agriculture (Table S1).

### 3.2. Phage Isolation and Characterization

**Figure 1:**
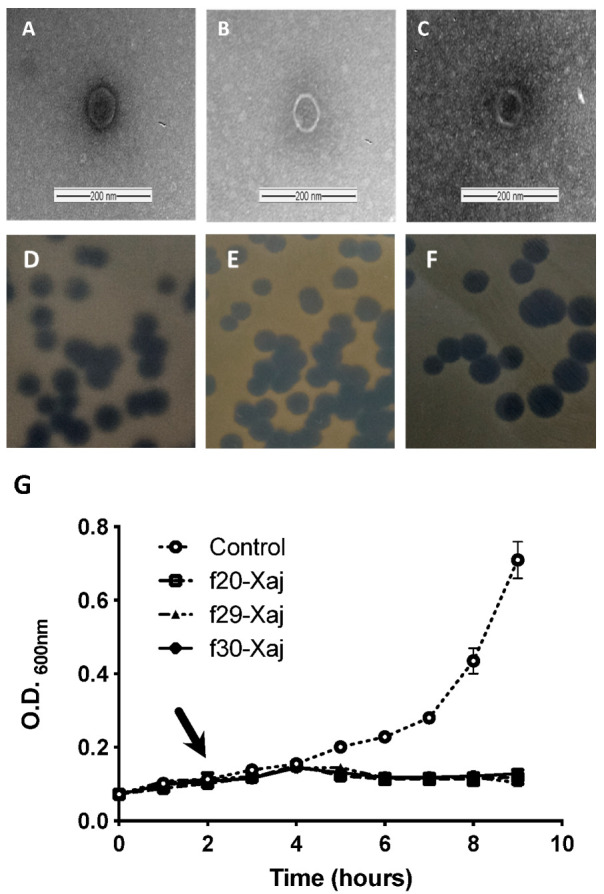
Morphological characterization of phages and in vitro lytic against *X. juglandis* J303. (**A**–**C**): MET, (**D**–**F**) Lysis plaque, (**G**) Delay curve. Arrow indicates the time of addition of individual bacteriophage. All experiments were employed in three independent replicates.

Twenty-three bacteriophages were isolated from translucent borders plaque of the *X. juglandis* J303 strain overlay culture, five phages from walnut tissue and eighteen from wet soil. From the in vitro lytic activity of isolated bacteriophages (Figure S2), three bacteriophages were selected for future trials based on their activity (Figure 1G) and host specificity against other strains from walnut tissue. These phages, denominated by *f*20-Xaj, *f*29-Xaj, and *f*30-Xaj, were characterized to generate a translucent/clear halo of 3 to 5 mm on the *X. juglandis* J303 isolate after 24–48 h of incubation (Figure 1D–F). The propagation of the bacteriophages using *X. juglandis* J303 in YPG broth reached titers close to 1 × 10^10^ PFU/mL, rising approximately one logarithm unit after precipitation with PEG-8000. Transmission electron microscopy revealed that all three-phage have a *Podovirus* morphology of the *Caudovirales* order with an icosahedral head (with sizes close to 50 nm in diameter) and short stubby tails (Figure 1A–C). All three phages selected showed lytic activity on fifteen *X. juglandis* isolated in this study and no apparent effect on other bacteria commonly present in the fruit and leaves of walnut crops (Table 1).

### 3.3. One-Step Curve of Bacteriophages

**Figure 2:**
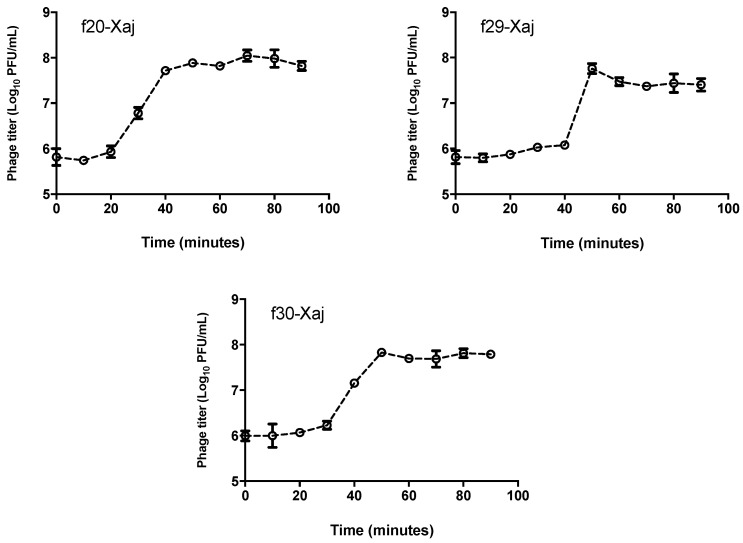
One-step growth curve for bacteriophages *f*20-Xaj, *f*29-Xaj, and *f*30-Xaj propagated in *X. juglandis* J303. The figure shows means ± standard error from three independent experiments.

The latent period and burst size were determined from the one-step growth curve, as a parameter of bacteriophages’ proliferation rates. In all three cases, the curves showed three phases, including the latent, rise, and plateau phases (Figure 2). The latent period of the phages *f*20-Xaj, *f*29-Xaj, and *f*30Xaj was 20, 40, and 28 min, respectively. However, the rise period noticeably decreases in *f*29-Xaj, taking only 10 min with respect to 20 min for *f*20-Xaj and *f*30-Xaj phages. The burst size of *f*20-Xaj, *f*29-Xaj, and *f*30-Xaj was 62.0 ± 15.7; 48.4 ± 5.3, and 57.9 ± 5.3 PFU per infected cell, respectively.

### 3.4. Lysogenic Assay and Phage Titer Stability

Different phage-resistant bacteria of *X. juglandis* J303 obtained from spots with the three bacteriophages used in this study (both individual or phage mix) showed no spot lysis plaque or signs of lysogeny using the spontaneous release technics. Furthermore, the PFU/mL behavior of *f*20-Xaj, *f*29-Xaj, and *f*30-Xaj phages under different treatments of temperature, and the liquid suspension was the same after twenty-eight days of incubation. Tap water, YPG culture medium, and YPG combined with algae extract are the best liquid substrates to maintain the bacteriophage titer independently at the treated temperature (Table 2). The bacteriophages’ titer stability suspended in distilled water decreases gradually as the temperature increases. However, in the presence of copper (commercial doses), the bacteriophage titer drops markedly, even at freezing temperatures.

### 3.5. Bioinformatics Analysis of the Bacteriophage Genomes

**Figure 3:**
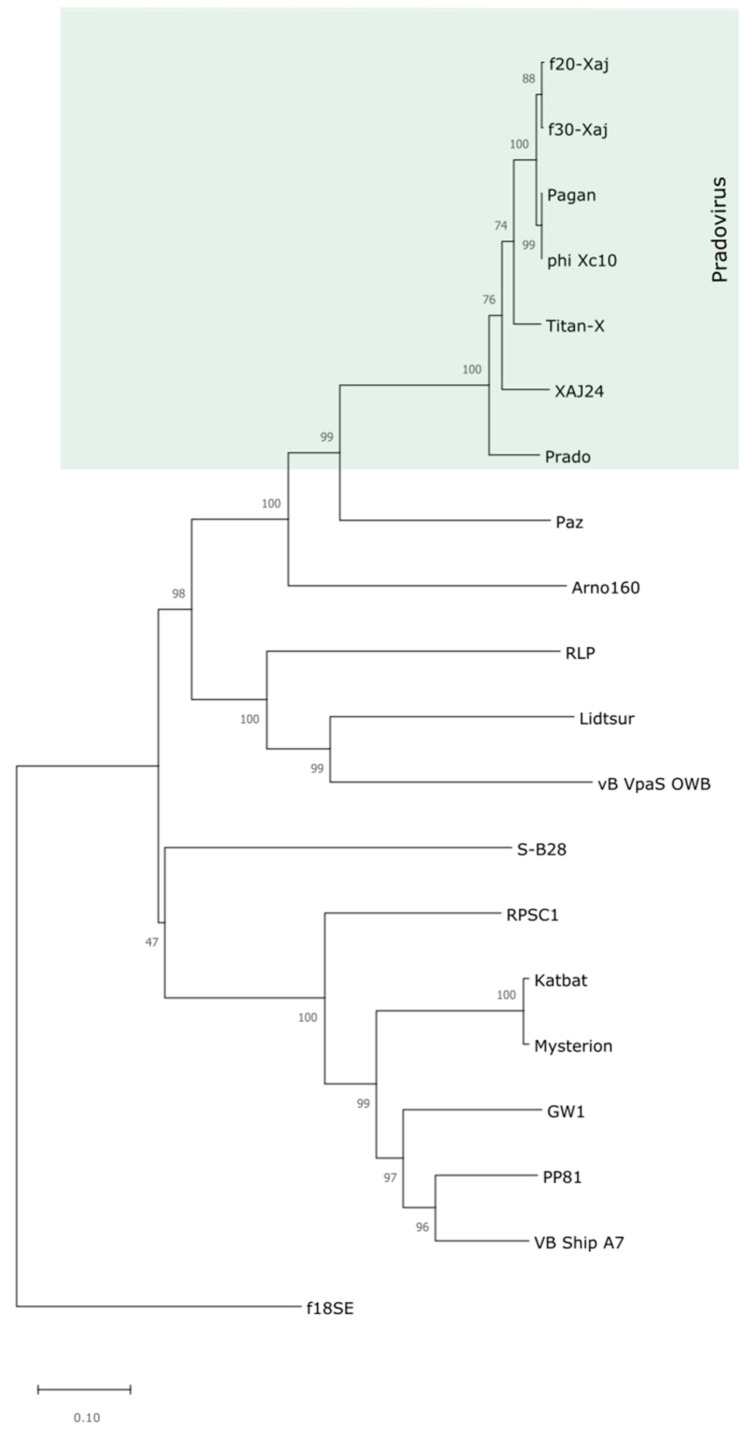
Phylogenetic tree of *Pradovirus* genus of bacteriophages made with whole-genome-based methodology through MEGAX software [39] and compared with other bacterial phytopathogen phages. The evolutionary history of *f*20-Xaj and *f*30-Xaj was inferred using the Neighbor-Joining method. The percentage of replicate trees in which the associated taxa clustered together in the bootstrap test (1000 replicates) are shown next to the branches [42].

Although all bacteriophages tested have the ability to lyse in vitro to Chilean and French isolates of *X. juglandis*, the bacteriophage *f*30-Xaj has a broader host profile and can lyse *X. corylina* obtained from hazelnut trees in Chile (Table 1). In this vein, the genomic analyses performed here included a bacteriophage with a strict host (*f*20-Xaj) and a broader host (*f*30-Xaj). Whole-genome-based phylogeny of both bacteriophages indicated that *f*20-Xaj and *f*30-Xaj are members of the *Pradovirus* phage, a genus within the subfamily *Autographivirinae* and family *Podoviridae* (Figure 3).

**Figure 4:**
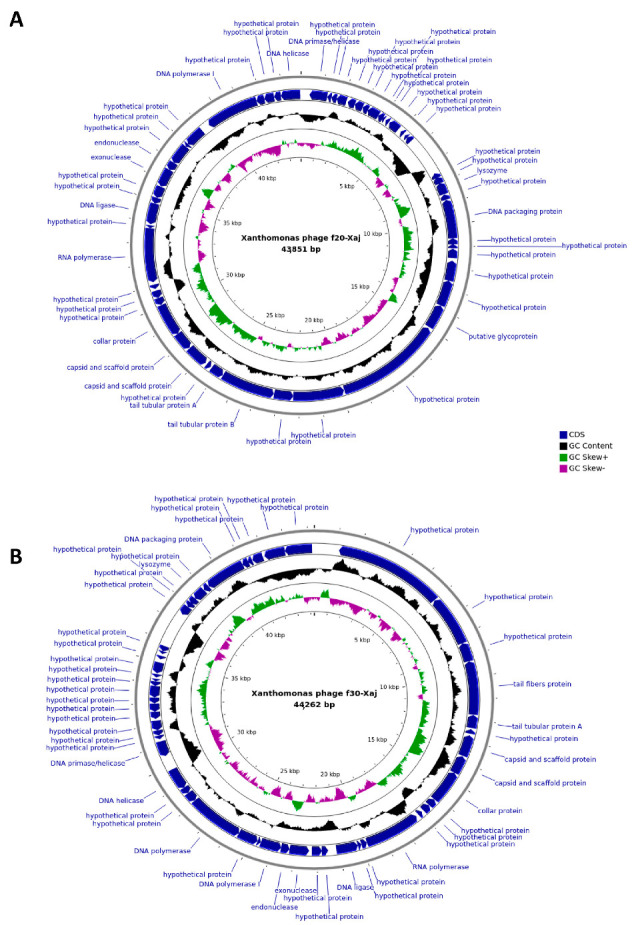
Map of genome organization of *f*20-Xaj (**A**) and *f*30-Xaj (**B**) bacteriophages using the BRIG platform and the CGView program. The ORFs are shown in blue arrows. The GC content is indicated by the black ring.

**Figure 5:**
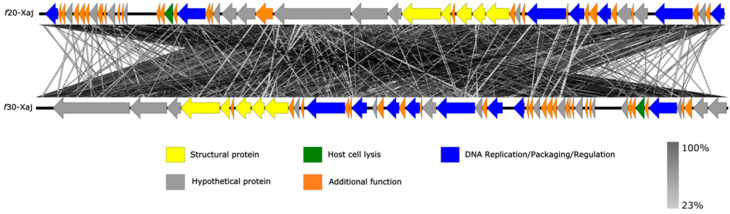
Schematic genomic alignment of *f*20-Xaj and *f*30-Xaj phage generated using the EasyFig program. Arrows with different colors represent ORFs according to their predicted functions indicated at the bottom. The grey bar in the lower right corner shows the identity percentage associated with the color of the bars connecting homologous regions.

Genome sequencing showed that the genome of *f*20-Xaj and *f*30-Xaj is circular and 43,851 and 44,262 bp in length, respectively (Figure 4). Similar open reading frames (ORFs) content was detected in both genomes, with 53 in *f*20-Xaj and 50 in *f*30-Xaj. None of the genomes analyzed contain temperate phage-associated genes such as encoding integrase, recombinase repressors, and/or virulence genes of pathogenic bacteria. To both bioinformatic predictions, the percentage of ORFs classified as hypothetical proteins (unknown function) was close to ~70%. Comparative analysis showed that *f*20-Xaj and *f*30-Xaj shared similar ORF content. However, one ORF-encoding putative glycoprotein that was present in *f*20-Xaj was absent in *f*30-Xaj (Figure 5).

**Figure 6:**
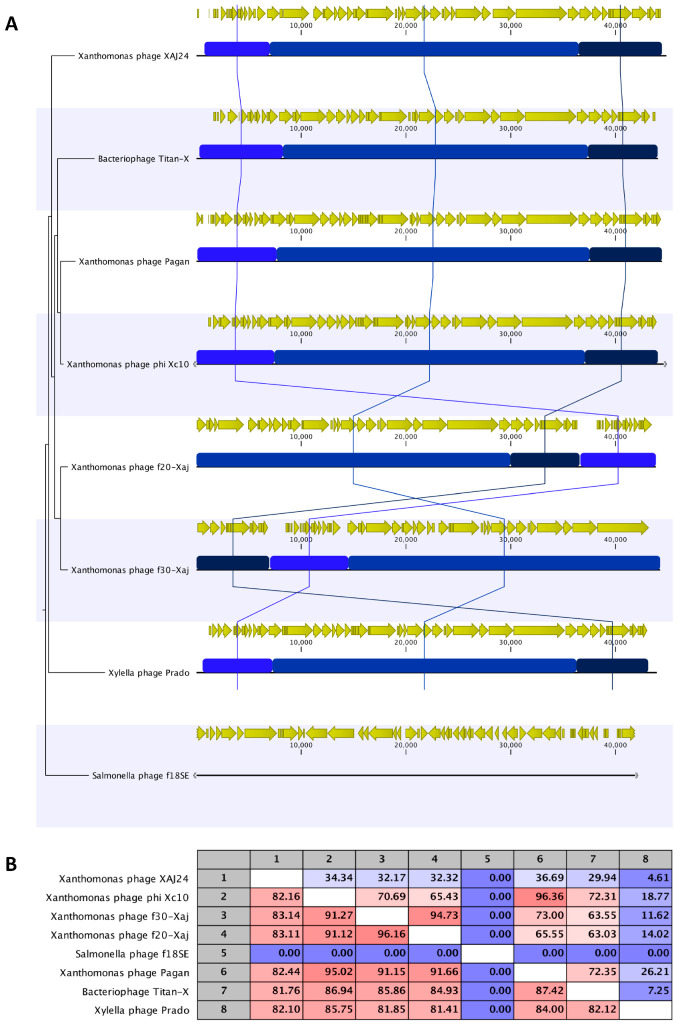
Genomic comparison at the nucleotide level of *f*20-Xaj and *f*30-Xaj genome to the other representative phages from the closest genus *Pradovirus*. (**A**) Whole-genome pairwise comparison of the eight phages’ complete nucleotide sequences, visualized as a matrix with percent identity with CLC Main Workbench 7. (**B**) ANI analysis table of eight *Pradovirus* phages selected. The color gradient from blue to red shows the percentage of identity, from lowest to highest, that each pair of phage genomes shares.

Although there are differences at the level of genomic organization between the different representatives of the *Pradovirus* group included in this study, including the *f*20-Xaj and *f*30-Xaj phages (Figure 6A), the nucleotide sequence identity is extremely high after performing ANI analysis (Figure 6B), reaching ranges of nucleotide identity from 81.4% to 96.16%. In this scenario, it is relevant to show that the genome of *f*20-Xaj and *f*30-Xaj phages share ~83% nucleotide identity with the previously reported phage Xaj24 with a lytic effect on *X. juglandis*. Particularly, the sequences of the tubular tail proteins of both bacteriophages share 98.75% identity; however, it is possible to identify a high-variability sequence zone in the putative tail of tubular protein A and a high level of single-nucleotide variations along the putative tail of tubular the protein B sequence (Figure S3A).

### 3.6. Walnut Field Trials Using Bacteriophages

**Figure 7:**
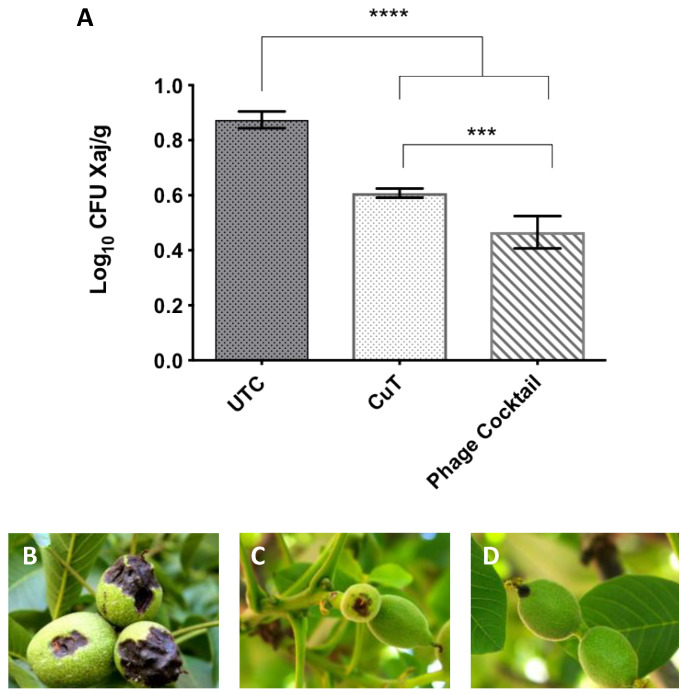
Reduction of bacterial load and walnut blight symptomatology in fruits obtained from treated fields. (**A**) Bacterial load (CFU/g) on walnut fruit post-treatments. UTC: Untreated control, CuT: Conventional treatment based on commercial copper compounds. Error bars represent standard deviation of three groups of tree walnut fruit from each treatment. Highly significant (****) different from the UTC (*p* < 0.0001); Significantly (***) different between groups. (*p* < 0.001). Representative images of immature walnut fruit post-treatment: (**B**) UTC, (**C**) CuT, and (**D**) phage cocktail (4 cc/L).

The application of copper sulfate significantly reduces the damage caused by *X. juglandis* on leaves compared to the control group (UTC). However, the highest dose of bacteriophage (4 cc/L) generated a comparable effect to copper treatment on both the incidence and severity of leaf damage associated with walnut blight (Table 3). A similar situation occurred in walnut fruit during the analyzed season. Bacteriophage applications on walnut trees reduced the incidence of walnut blight in immature fruit during the period from November 2015 to February 2016 without showing statistical differences with respect to conventional treatment (CuT). In this same period, the severity in immature walnut fruits after the application of bacteriophages developed a lower severity degree compared to the UTC group, regardless of the dose applied. In this sense, only the application with the highest dose of phages (4 cc/L) presented a similar statistical value to the conventional treatment (*p* < 0.05). The immature fruits analyzed showed no visual differences in the apical area of necrotic damage after the application of phage or CuSO_4_ treatments (Figure 7B–D). Although the degree of severity differs statistically in the last period evaluated (February 2016) between the highest dose of bacteriophages applied and the conventional treatment (CuT), the load of *X. juglandis* in these tissues is strongly reduced by the use of these biological entities (Figure 7A). Applications of high doses of bacteriophages (>3 cc/L) increase walnut fruit production with respect to the UTC group. However, these applications present significant differences with respect to conventional treatment (Table 3). None of the bacteriophage treatments showed symptoms of phytotoxicity at neither the leaf nor fruit level during all periods analyzed in the field.

## 4. Discussion

Synthetic chemical compounds are commonly used to treat crop diseases, despite the knowledge that they facilitate the emergence of resistant phenotypes of pathogens as well as restrictions on their use due to toxicity problems. The use of effective environmentally friendly control alternatives makes bacteriophages a promising alternative for agricultural uses [43]. The resistance to both copper and some antibiotics reported in different isolates of the *Xanthomonas* group, and *X. juglandis*, reinforces the idea of generating alternative strategies to reduce the incidence of these pathogens, with the use of bacteriophages being an option [44]. There are some studies reporting the isolation and characterization of bacteriophages against *X. juglandis* [16,17,18]. However, none of them demonstrate their usefulness in the control of this pathogen on plant tissue.

To isolate and propagate bacteriophages, we used the *X. juglandis* J303 strain due to prior knowledge of its genome [11] and its ability to generate necrotic damage on an in vitro immature walnut fruit pathogenicity assay. This bacterial strain showed a similar biochemical profile to strain NCPPB 411 and Romania or Hungary isolates [45]. The strain *X. juglandis* J303 is susceptible to different antibiotics and commercial doses of copper sulfate, and there is a wide range of resistance profiles between the Chilean and French isolates of *X. juglandis* obtained in this study (data not shown). On the other hand, the bacteriophages *f*20-Xaj, *f*29-Xaj, and *f*30-Xaj have lytic activity against different *X. juglandis* isolates regardless of their resistance profile, the origin of isolation, and even against some closely related species such as *X. corylina*. However, the use of molecular markers to determine the identity of isolates such as *X. juglandis* is essential to extend the specific host range of these bacteriophages against this phytopathogen [46]. Furthermore, the characterization of different populations of *X. juglandis* in the field is a determinant of the success and inclusion of a phytosanitary program based on bacteriophages against walnut blight. This is justified by possible variations in the external protein structures of these bacteria that could decrease the effectiveness of recognition by bacteriophages, in addition to the evidence of the existence of different pathovars of *Xanthomonas* in the same tree [47].

The morphology of viral particles and genome analysis showed that *f*20-Xaj and *f*30-Xaj belongs to the *Pradovirus* genus. No phages were recovered from supernatants from bacteria that resisted bacteriophage infection in vitro. The lytic phenotype observed from these bacteriophages is consistent with the absence of genes associated with lysogeny in the genomes of the selected bacteriophages. The high specificity shown by selected bacteriophage suggests that bacterial isolates of *X. juglandis* obtained from Chile and France share ligands for the recognition of these viral entities [48]; however, further studies are needed. In this scenario, genes that encode for a putative tail tubular protein from *f*20-Xaj and *f*30-Xaj present single-nucleotide polymorphisms that can modify protein translation (Figure S3B), thus their ability to recognize the host [49] may partly account for the differences in their host range. This is a key aspect to consider in order to generate bacteriophage cocktails with a broad spectrum against variant populations of *X. juglandis*.

The *f*20-Xaj, *f*29-Xaj, and *f*30Xaj bacteriophages reduce *X. juglandis* cell density at a lower MOI and approximately 50% less in the period (minutes) of latency than previously reported bacteriophages Xaj2 and Xaj24 from Hungary [18]. Although, the differences detected between these two groups of bacteriophages could be influenced by the type of host cell used and the culture conditions employed in each infection kinetic assay. The *f*20-Xaj, *f*29-Xaj, and *f*30-Xaj phages appear to be promising candidates as biocontrol agents against *X. juglandis*. Electron microscopy analysis showed the production of a matrix of extracellular polymeric substances (EPS) by *X. juglandis* J303 similar to that previously reported in *Xanthomonas arboricola* pv. *pruni* [50].

To date, there are few studies that address the effectiveness of the use of bacteriophages in field [14]. Most of these studies have driven bacteriophage characterization towards stability parameters of these viral particles (PFU/mL) against various physicochemical parameters such as temperature, pH, UV radiation, and compatibility with agrochemicals. Although some of them use plant models under controlled conditions (laboratory and/or greenhouse), field trials present a technical difficulty in determining the effectiveness of bacteriophage usage or any other biocontrol agent. In this study, the sampling design of the groups of trees presented a historical record of walnut blight, and only the natural load of *X. juglandis* was considered in the treatment.

According to our results, the selected phages, in a high dose (>4 cc/L), showed a comparable effect to the conventional use of copper (CuSO_4_) on the reduction of symptomatology caused by *X. juglandis* infection in both walnut leaves and fruit. The bacteriophage applications in this study showed no effects attributable to phytotoxicity. Although the algae extract may favor the stability of the titers on tested bacteriophages (PFU/mL), a final formulation with these active biological principles should be subject to further analysis in terms of efficacy, phytotoxicity, long-term stability, and/or improvement in performance [51]. The damage observed in immature walnut fruit after the application of bacteriophages and copper was associated with a reduced zone of apical necrosis. In this sense, and in addition to the high specificity shown by bacteriophages, it is relevant to include, in future determinations, the presence of fungi and/or bacteria resistant to agrochemicals capable of generating damage specifically in these areas, a condition known as Brown Apical Necrosis (BAN) [52], which could justify the differences observed in fruit yield in the trees analyzed.

In summary, the incorporation of a bacteriophage-based biocontrol strategy must demonstrate effectiveness in field conditions of agricultural production and add to the knowledge of compatibilities with products commonly used in agriculture, in order to begin the process of obtaining legal authorizations from the respective regulatory entities [15]. To our knowledge, this is the first report where the parameters of effectiveness of bacteriophage applications in the biocontrol of walnut blight caused by *X. juglandis* are determined under field conditions.

## Figures and Tables

**Table 1:** Bacteriophage specificity to different bacteria isolated from walnut trees. The bacterial identification was generated by sequencing the 16S rRNA portion or specific genes.

Bacterial Strains	Biological and Geographical Origin	Year Isolation	Bacteriophages		
			*f*20-Xaj	*f*29-Xaj	*f*30-Xaj
*X. arboricola* pv. *juglandis* J303	Walnut, VIII Region-Chile	2011	+	+	+
*X. arboricola* pv. *juglandis* N3	Walnut, VIII Region-Chile	2014	+	+	+
*X. arboricola* pv. *juglandis* 3A	Walnut, VIII Region-Chile	2014	+	+	+
*X. arboricola* pv. *juglandis* 3BT	Walnut, VIII Region-Chile	2015	+	+	+
*X. arboricola* pv. *juglandis* LMM13	Walnut, VIII Region-Chile	2015	+	+	+
*X. arboricola* pv. *juglandis* LMM14	Walnut, VIII Region-Chile	2015	+	+	+
*X. arboricola* pv. *juglandis* GN2	Walnut, RM-Chile	2016	+	+	+
*X. arboricola* pv. *juglandis* GN3	Walnut, RM-Chile	2016	+	+	+
*X. arboricola* pv. *juglandis* GN4	Walnut, RM-Chile	2016	+	+	+
*X. arboricola* pv. *juglandis* GN5	Walnut, RM-Chile	2016	+	+	+
*X. arboricola* pv. *juglandis* FS11	Walnut, Toulouse-Francia	2018	+	+	+
*X. arboricola* pv. *juglandis* FS12	Walnut, Toulouse-Francia	2018	+	+	+
*X. arboricola* pv. *juglandis* FS15	Walnut, Toulouse-Francia	2018	+	+	+
*X. arboricola* pv. *juglandis* FS17A	Walnut, Toulouse-Francia	2018	+	+	+
*X. arboricola* pv. *juglandis* FS18	Walnut, Toulouse-Francia	2018	+	+	+
*X. arborícola* pv. *corylina* H.1	Hazelnut, VII Region-Chile	2016	−	(+)	+
*X. arborícola* pv. *corylina* H1.3	Hazelnut, VII Region-Chile	2016	−	(+)	+
*X. arborícola* pv. *corylina* H.2	Hazelnut, VII Region-Chile	2016	−	−	+
*X. arborícola* pv. *corylina* H1.4	Hazelnut, VII Region-Chile	2016	−	−	+
*Xanthomonas campestris*	Hazelnut, VII Region-Chile	2015	−	−	−
*Pantoea agglomerans*	Walnut, VIII Region- Chile	2014	−	−	−
*Curtobacterium flaccumfaciens*	Walnut, VIII Region- Chile	2015	−	−	−
*Brachybacterium paraconglomeratum*	Walnut, VIII Region- Chile	2015	−	−	−
*Leucobacter tardus*	Walnut, VIII Region- Chile	2015	−	−	−
*Enterobacter* sp.	Walnut, VIII Region- Chile	2015	−	−	−
*Bacillus cereus*	Walnut, VIII Region- Chile	2015	−	−	−
*Agrobacterium tumefacien* ^1^	Plum, RM-Chile	2017	−	−	−

(−) No plaque; + clear plaque formation; (+) turbid plaque formation; ^1^ strain donated by Servicio Agricola y Ganadero (SAG-Chile).

**Table 2:** Titer stability of phages under different liquid suspensions and temperatures 28 days post-incubation. This table gathers the results obtained from the three bacteriophages evaluated independently.

Temperature (°C)	Distilled Water	Tap Water	YPG Broth	YPG Broth + Algae Extract	YPG Broth + Cuprous Oxide	YPG Broth + Copper Hydroxide	YPG Broth + Copper Sulfate
−20	+++	+++	+++	+++	+	+	++
4	++	+++	+++	+++	−	−	+
17	+	+++	+++	+++	−	−	−
28	−	+++	+++	+++	−	−	−

(+++) Maintains titer of phage to 1 × 10^5^ PFU/mL; (++) decrease of 1 log unit PFU/mL; (+) decrease of 2 log unit PFU/mL; (−) decrease of more than 3 log unit PFU/mL.

**Table 3:** Evaluation of incidence and severity in fruit and leaves of walnut trees treated with bacteriophages mix.

		Leave		Fruit						Performance (Kg)
		Incidence (%)	Severity (Grade)	Incidence (%)	Severity (Grade)	Incidence (%)	Severity (Grade)	Incidence (%)	Severity (Grade)	
**Treatments**		**November 2015**		**November 2015**		**January 2016**		**February 2016**		**March 2016**
**UTC**		66.3 ^b^	3 ^b^	38.75 ^c^	3 ^b^	58.13	4 ^c^	37.5 ^b^	4 ^c^	10.25 ^a^
**CuT**		25.6 ^a^	2 ^a^	8.75 ^bc^	2 ^a^	12.5	2 ^a^	22.5 ^a^	2 ^a^	25 ^c^
**Phage cocktail**	2 cc/L	41.88 ^c^	2^a^	33.13 ^abc^	2 ^a^	46.25	3 ^b^	30 ^ab^	3 ^b^	12.75 ^a^
	3 cc/L	39.38 ^bc^	2^a^	28.75 ^ab^	2 ^a^	40	3 ^b^	29.38 ^ab^	3 ^b^	17.0 ^b^
	4 cc/L	30.0 ^ab^	2 ^a^	11.88 ^a^	2 ^a^	22.5	2 ^a^	28.13 ^a^	2.75 ^b^	19.75 ^b^

UTC: Untreated control, CuT: Conventional treatment based on commercial copper compounds. Averages in the same column with the same letter do not differ statistically (*p* < 0.05). The dates cited in the table indicate the times of sampling from the treated trees.

## Data Availability

All data supporting the conclusions of this article are included in this article.

## Supplementary Materials

The following supporting information can be downloaded at: https://www.mdpi.com/article/10.3390/v14071380/s1, Figure S1: Characterization of *Xanthomonas arboricola* pv. *juglandis* J303 employed for isolation and propagation of bacteriophages.; Figure S2: In vitro activity assay of bacteriophages against *Xanthomonas arboricola* pv. *juglandis* J303; Figure S3: Alignment analysis of tail tubular protein of *f*20-Xaj and *f*30-Xaj bacteriophages; Table S1: Biochemical and antibiotic resistance profile of *Xanthomonas arboricola* pv. *juglandis* strain J303.
