# Supplementary material for: Characterization of Xanthomonas arboricola pv. juglandis Bacteriophages against Bacterial Walnut Blight and Field Evaluation
Source: Viruses. 2022 Jun 24;14(7):1380. doi: 10.3390/v14071380 (PMC9325289; doi:10.3390/v14071380)
Supplement: Supplementary file 1 [file viruses-14-01380-s001.zip › viruses-1686173-supplementary.pdf]

Supplementary Materials

Table S1. Biochemical and antibiotic resistance profile of *Xanthomonas arboricola* pv. *juglandis* strain J303 employed in this study.

**Reaction/Enzyme**	Xaj J303 WT
Reduction of nitrates to nitrites	-
Reduction to nitrates to nitrogen	+
Indole production	-
Glucose fermentation	-
Arginine Dihydrolase	+
Urease	+
Esculin hydrolysis	+
Gelatin hydrolysis	+
B-galactosidase	+
**Carbohydrate assimilation**	
Glucose	+
Arabinose	-
Mannose	+
Mannitol	-
N-Acetyl-Glucosamine	+
Maltose	-
Potassium Gluconate	-
Capric Acid	-
Adipic Acid	-
Malate	+
Trisodium Citrate	+
Phenylacetic Acid	-
**Antibiotic resistance profile (mm)**	
Gentamicin	22 ± 0,2
Streptomycin	32 ± 0,7
Oxytetracycline	37 ± 0,5

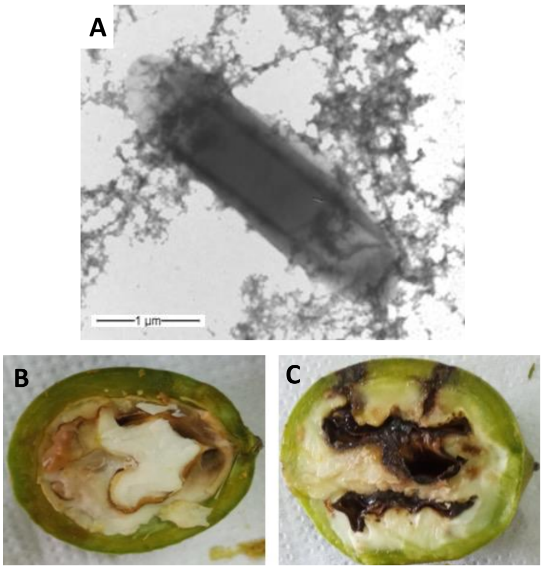

Figure S1. Characterization of *Xanthomonas arboricola* pv. *juglandis* J303 employed for isolation and propagation of bacteriophages. (A) Transmission electron microscopy of *X. juglandis* strain J303 (bar 1 µm). Necrotic damage in immature walnut fruit: Control PBS (B) and *X. juglandis* J303 (C).

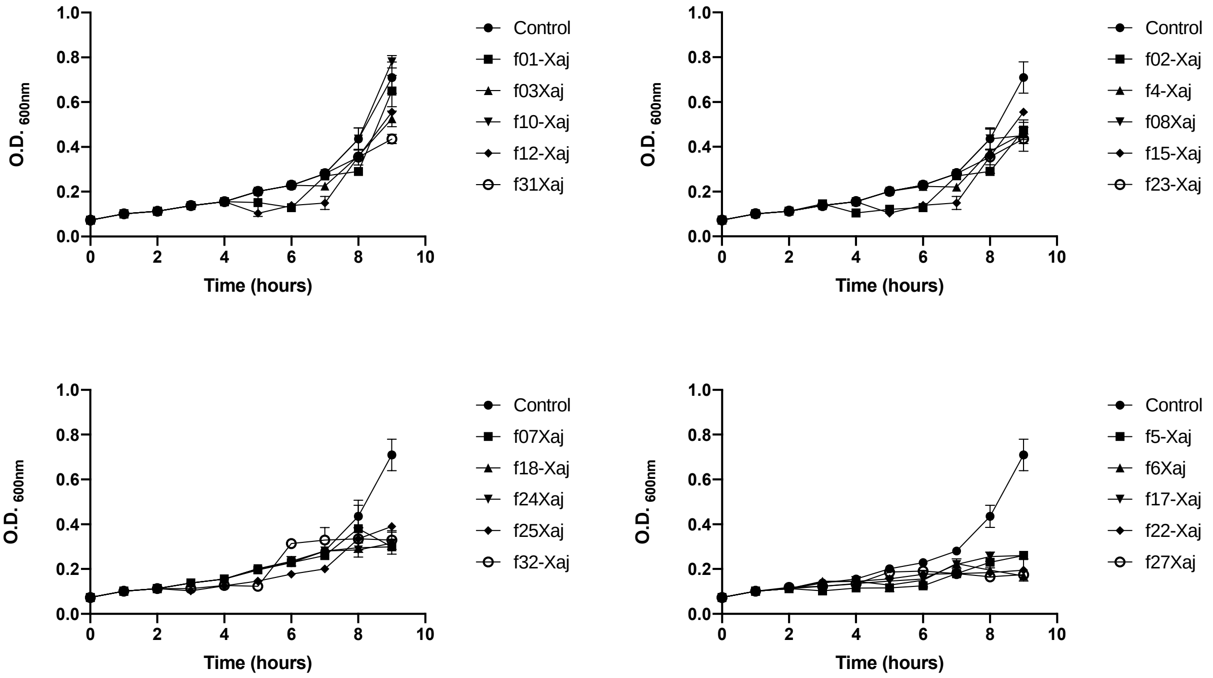

Figure S2. In vitro activity assay of bacteriophages against *Xanthomonas arboricola* pv. *juglandis* J303. The phages were grouped in each graph according to the OD600nm ranges they reached at the end of the assay. All experiments were employed in three independent replicates.

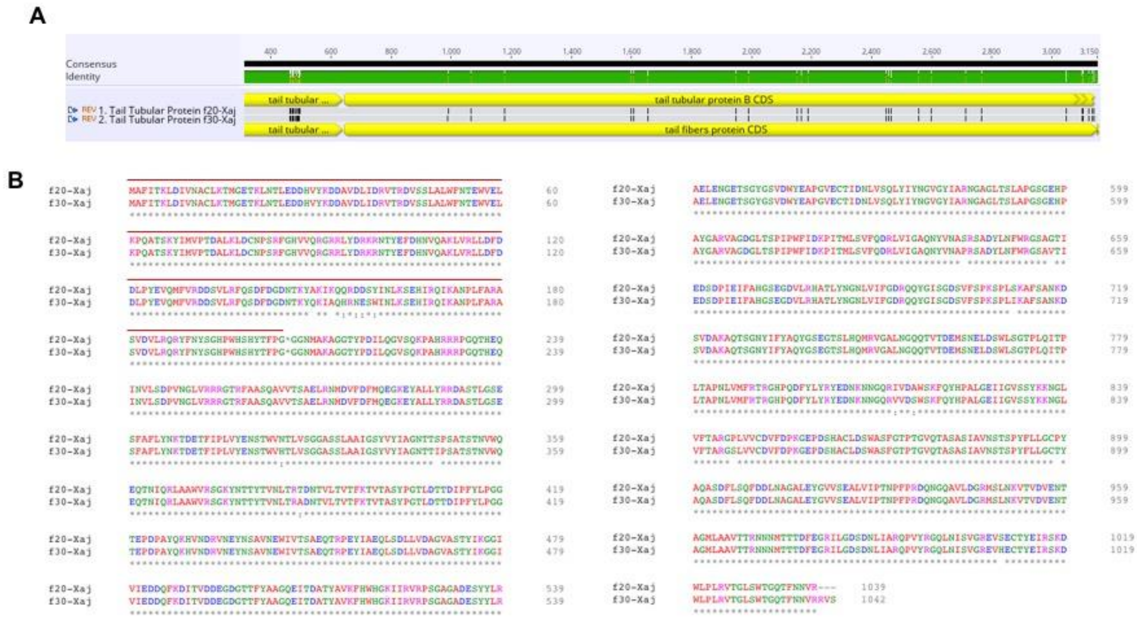

Figure S3. Alignment analysis of tail tubular protein of *f*20-Xaj and *f*30-Xaj bacteriophages. (A) Schematic depicting nucleotide changes between sequences performed with Geneious Prime® 2022.0.2 software. (B) Alignment of putative bacteriophage tail tubular proteins A and B performed with online Clustal-Omega server. Only the amino acid sequence of tubular protein A is indicated with the upper red line.
